# Students' acceptance of digital media in physical education—a cross-sectional study

**DOI:** 10.3389/fspor.2026.1654602

**Published:** 2026-02-09

**Authors:** Juliane Mackenbrock, Jens Kleinert

**Affiliations:** Section Health & Social Psychology, Institute of Psychology, German Sport University Cologne, Cologne, Germany

**Keywords:** adolescents, children, digital media, motivation, physical education, self-determination theory, technology acceptance, technology integration

## Abstract

**Background:**

The use of digital media (DM) in physical education (PE) is increasing. However, students' acceptance of DM in PE is unclear. The Organismic Integration Theory as part of the Self-Determination Theory serves as a theoretical framework to discuss students' acceptance.

**Objective:**

The study aims are 1) to determine the strength of students' acceptance of DM use, 2) to analyze differences in acceptance of different types of DM use, and 3) to identify predictors of DM acceptance in PE.

**Methods:**

A cross-sectional study with 291 German secondary students (female = 153; male = 138; *M*_age_ = 15.78 years, *SD* = 1.13) was conducted. The data were collected with an online-questionnaire consisting of demographic data, DM usage during leisure time and at school, usage intention for different types of DM use, behavioral regulation regarding future DM use, and barriers/reasons against DM use in PE.

**Results:**

1) Descriptive analysis of the strength of acceptance showed moderate DM use intention for instruction, feedback, organization, and knowledge transfer as well as high usage intention for entertainment. 2) An analysis of variance indicated significant differences in the acceptance of the different types of DM use ($\eta_{p}^{2}=.023$), with the covariates of age ($\eta_{p}^{2}=.017$) and gender ($\eta_{p}^{2}=.025$) playing a role in some types of DM use. 3) In a multiple linear regression explaining 51% of the variance, identified regulation (i.e., usefulness) (ß = .34) and intrinsic motivation (i.e., enjoyment) (ß = .26) were identified as positive predictors of DM acceptance, whereas introjected regulation (i.e., social norms/expectations) (ß = −.11) and amotivation (i.e., meaninglessness) (ß = −.16) were negative predictors.

**Conclusion:**

Overall, students' acceptance of the different types of DM use in PE was moderate. Only entertainment showed a strong acceptance probably due to the students' previous experiences. In line with previous technology acceptance research in educational contexts, usefulness and enjoyment were found to be relevant predictors of DM acceptance. The differences in acceptance of different types of DM use in PE and the relevance of usefulness and enjoyment, should be considered by PE teachers for the successful integration of DM. Future research should investigate differences in DM acceptance in other populations (e.g., primary education), and longitudinal studies are needed to examine causal effects.

## 1 Introduction

The use of digital media (DM) such as smartphones, tablets, and other visual display media and corresponding applications (1) in physical education (PE) is increasing (2, 3). Recent literature reviews have indicated that the use of DM in PE can be associated with positive outcomes such as improved movement learning (2) or enhanced motivation (4, 5). However, the use of DM in PE needs acceptance by teachers and students (6). While teachers' acceptance of DM in schools, especially in PE, has been examined in several studies [e.g., (7–10)], comparatively little is known about students' acceptance of DM in PE, although students are in many cases of DM use in PE the main users. Therefore, it is important to investigate students' perspective on DM in PE. Accordingly, the rationale of this study is to gain knowledge about how DM in PE are accepted by students and under which circumstances they are motivated to use DM in PE.

The Organismic Integration Theory (OIT) provides a theoretical framework to examine students' motivation to use DM in PE. The OIT is a mini-theory of Deci and Ryan's Self-Determination Theory [SDT: (11)], which explains motivational processes in human behavior. According to the OIT, behavior can be amotivated, extrinsic, or intrinsic and is arranged on a continuum from no self-determination to full self-determination (12, 13). Amotivation can be described as a lack of behavioral intention and absence of self-determination in behavior. Extrinsic motivation can be differentiated into four types of behavioral regulation: external, introjected, identified, and integrated regulation. External regulation is a type of behavioral regulation in which behavior is controlled by external aspects like rewards or punishments. Introjected regulation consists of behavior which is mainly lead by social norms. Identified regulation focuses on rational beliefs regarding the behavior. Integrated regulation refers to internal and personal values that align with the identity. As the most self-determined type of behavioral regulation, intrinsic motivation is characterized by its focus on the behavior itself (e.g., pleasure and interest) and not on its consequences (12, 13).

The motivation to use DM in school is mostly described in relation to technology acceptance, which is seen as “a key factor for successful introduction and intended use of new technology” [(14), p. 11]. Research on DM acceptance is mainly based on the Technology Acceptance Model (TAM) by Davis (15, 16). At its core, the TAM includes the factors of perceived usefulness and perceived ease of use, which influence first behavioral intentions for technology use and second actual technology use (17). Over the years, several versions of the TAM have been developed. Although they have remained the same in essence, they include additional variables such as subjective norm and voluntariness (TAM-2) or perceived enjoyment (TAM-3) (18).

**Figure 1:**
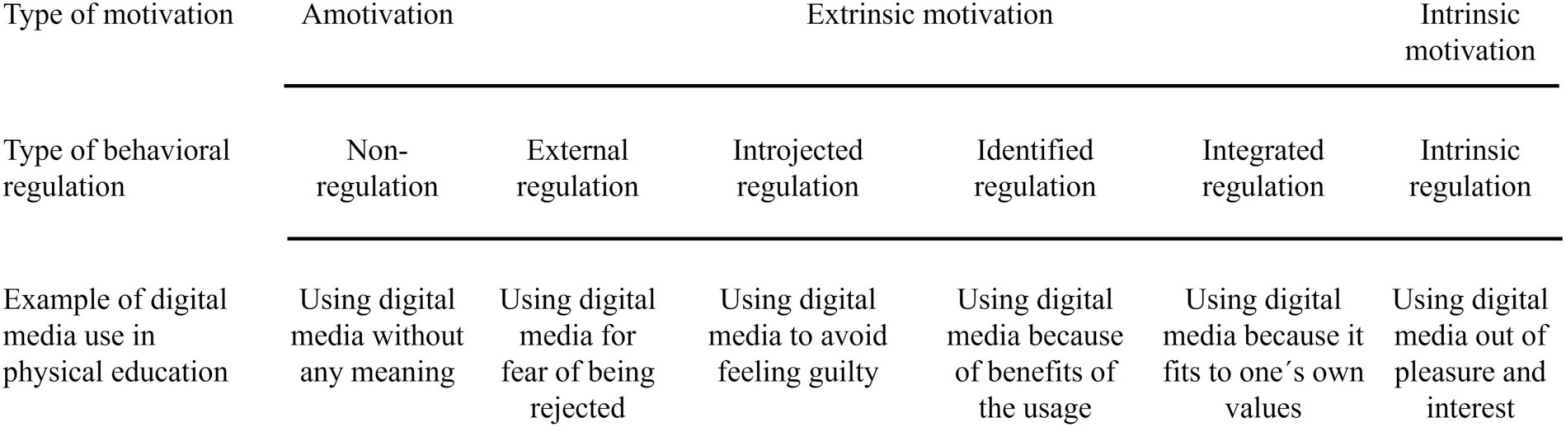
Continuum of behavioral regulation regarding the use of digital media in physical education (adapted from Deci & Ryan, 2000, p. 237).

The OIT and the TAM are bridged as the types of behavioral regulation (i.e., OIT) reflect aspects of TAM and its extensions. In more detail, the TAM includes aspects of extrinsic regulation (e.g., perceived usefulness, subjective norm) which are highly connected to types of behavioral regulation in OIT (e.g., perceived usefulness reflected in identified regulation; subjective norm reflected in introjected regulation). Moreover, the TAM [respectively the TAM extensions; (18)] contains aspect of intrinsic behavioral regulation (e.g., perceived enjoyment reflected in intrinsic motivation). Despite this conceptual overlap between OIT and TAM, the explanatory strength of OIT is in particular based on its theoretical consistency in explaining motivational processes in physical education (19), whereas TAM provides a context specific framework involving different motivational elements from different basic theories [e.g., Theory of Planned Behavior; (15, 16)]. Overall, the OIT as a popular and suitable theory used in research on students' motivation in PE (19) provides an opportunity to conceptualize different types of behavioral regulation of students' regarding the use of DM in PE (see Figure 1). These types of behavioral regulation serve to explain behavioral intention, which in the narrow sense can be understood as acceptance (14).

The research on technology acceptance in educational settings is extensive. Especially in the field of higher education, there are many original studies on different DM [e.g., e-learning platforms: (20); chat-bots: (21)] summarized in several literature reviews (22–25). Overall, these reviews have shown that the core elements of perceived usefulness and perceived ease of use are the most researched factors (22, 25) and that they often have strong correlations with behavioral intention to use DM [e.g., (26)]. Besides these factors, other motivational aspects such as perceived enjoyment and self-efficacy play a less researched but nevertheless relevant role in explaining students' DM acceptance by predicting the core elements of perceived usefulness and perceived ease of use (22, 27, 28). Furthermore, barriers against the use of DM, such as the complexity of the DM, have been identified as factors that negatively influence behavioral intention for the use of DM (25).

Although studies on technology acceptance in educational settings have mainly focused on higher education, some studies have focused on DM acceptance among primary and secondary school students [e.g., (29–33)]. These studies have shown that, in addition to the relevance of perceived usefulness, perceived ease of use, and perceived enjoyment in the acceptance of DM in PE, gender differences were found: Boys are more likely to accept DM in educational settings than girls, as they use DM more frequently in their free time (29, 33).

Research on students' acceptance of DM in PE is limited compared to research on DM acceptance in the educational sector in general. The studies on students' acceptance of DM in PE vary in terms of study results, theoretical background, DM use, study group, and research methods. Regarding the study results, students see both positive [e.g., improved movements and tactics: (34, 35)] and negative aspects [e.g., technical issues and reduced physical activity time: (35); less interaction with PE teachers: (36)] of DM use in PE. Concerning the theoretical foundation, the predominant technology acceptance models are also used in PE [e.g., TAM-based questionnaire: (37, 38); deductive interview technology acceptance factors: (36)]. In the studies of Greve et al. (34) and Koh et al. (35), no theoretical background on technology acceptance was presented. With regard to the use of DM, Greve et al. (34) and Koh et al. (35) looked at the use of tablets in PE, Killian and Woods (36) investigated an online course with theoretical aspects of PE, Merino-Campos et al. (37) analyzed video games to improve basketball skills and Roth et al. (38) focused on students' attitudes toward videos in PE. Four studies focused on secondary education students [10–18 years old: (35–38)], and one focused on primary education students [5–10 years old: (34)]. Three of the studies used qualitative methods (34–36), and two used quantitative methods (37, 38).

The research on the acceptance of DM by students in PE is insufficient for several reasons. First, compared to the educational context in general, only few studies focusing on PE have been conducted. Second, studies focusing on PE have provided heterogeneous results on students' DM acceptance. Third, recent research has mainly referred to specific examples of DM use that students have encountered in their PE lessons (e.g., apps for motion analysis), neglecting the general acceptance of DM in PE (i.e., students' intention to use DM in PE) and lacking comparisons between different types of DM use.

Therefore, the purpose of our study is to expand the research on students' acceptance of DM in PE. We conducted a quantitative cross-sectional study with students in PE. Within our study, we looked at different types of DM use based on a classification from Mackenbrock and Kleinert (4) (e.g., DM for instruction, DM for entertainment) and asked students about their usage intention—a key factor of technology acceptance (14, 16)—for different types of DM use in PE. We used the OIT as a theoretical framework because it is an adequate framework for integrating aspects of technology acceptance into the context of students' motivation in PE (19, 39). Therefore, our study examines the different forms of behavioral regulation mentioned in the OIT (i.e., amotivation, external regulation, introjected regulation, identified regulation, integrated regulation, and intrinsic motivation) regarding the use of DM in PE.

In light of the research gap identified above and our theoretical considerations, we aimed to answer the following research questions (RQs):

RQ1: How strong is students' acceptance of different types of DM in PE (i.e., intention for future use)?

RQ2: Does students' acceptance of DM in PE (i.e., intention for future use) differ between the different types of DM use?

We hypothesize that students' acceptance of DM in PE (i.e., intention for future use) differs significantly between different types of DM use in PE.

RQ3: Which type of behavioral regulation regarding the use of DM in PE explains students' acceptance of DM in PE (i.e., the intention for future use) in which amount?

We hypothesize that different types of behavioral regulation significantly explain students' acceptance of DM in PE (i.e., intention for future use), whereby self-determined types of behavioral regulation being positive predictors and controlled types negative predictors.

## 2 Materials and methods

### 2.1 Participants

Students in grades 9–11 at German secondary schools (14–18 years old) were eligible to participate in the study. A total of 299 students from seven schools in North Rhine-Westphalia and Schleswig-Holstein participated voluntarily in the study. Students without information on gender or whose gender was something other than male or female (*n* = 5) were excluded from the analyses, as the small sample size precluded statistical comparisons with male and female groups. Moreover, participants outside the age span (*n* = 3) were excluded. The final sample consisted of 291 students with a mean age of 15.78 years (*SD* = 1.13), of which 153 were female and 138 were male. Whereas 277 of the participants attended a grammar school, 14 attended a comprehensive school in grade 11 (i.e., equivalent qualification as at a grammar school).

### 2.2 Measures

#### 2.2.1 Sociodemographic variables

Students were asked to answer sociodemographic questions regarding their age, gender, school type, and class level.

#### 2.2.2 Digital media use during leisure time and at school

To obtain an overview of the current DM usage behavior of the sample, self-developed questions on DM usage behavior were asked. The questions related to two sub-areas: 1) DM use during leisure time (amount of screen time, frequency of used devices, used apps, and usage purposes) and 2) DM use at school (frequency of DM use for different school subjects, frequency of devices used in PE, and DM usage purposes in PE). Background information describing the sample in their DM use during leisure time and at school can be found in Section 3.

#### 2.2.3 Intention for future DM use

Acceptance was operationalized in a narrow sense as students' intention for future DM use in PE. The intention for future use of five different types of DM use was measured with one item per type of use in PE to analyze differences between the types. The instruction was as follows: “Would you like digital media to be used in PE for the following purposes in the future?” The different types of DM use were explained with short examples, such as “providing feedback/correction (e.g., filming your own exercise execution)”. Answers were recorded using a 6-point Likert scale ranging from 1 (definitely not) to 6 (definitely). For the regression analysis, the five types of DM use were subsumed under an overall DM use intention score (Cronbach's *α* = .74). An exploratory factor analysis supported a unidimensional structure of intention to use for the five types of DM use in PE (KMO = .753; Bartlett´s Test Chi^2^(10) = 330.22, *p* < .001). Both the eigenvalue (2.50) and the scree plot indicated one factor explaining 50% of the variance, justifying the aggregation into an overall DM-use intention score.

#### 2.2.4 Behavioral regulation of the use of DM

Students' behavioral regulation of the use of DM in PE was assessed using a 12-item short version of the behavioral regulation questionnaire [BRQ-12: (40)]. The BRQ-12's twelve items represent the six types of behavioral regulation according to Deci and Ryan (11, 12), where each type is represented by two items. Every item followed the stem “I would use digital media in physical education, …”: amotivation (e.g., “… but I wouldn’t know why.”); external regulation (e.g., “… because I would be forced to.”); introjected regulation (e.g., “… because it would be required of me.”); identified regulation (e.g., “… because it would be useful for me.”); integrated regulation (e.g., “… because it would suit me well.”); intrinsic motivation (e.g., “… because I would enjoy it.”). The answers were recorded using a 6-point Likert scale ranging from 1 (does not apply at all) to 6 (fully applies). Confirmatory factor analysis (CFA) was conducted to examine the BRQ-12 six-factor structure. The model showed a good fit, as indicated by the fit indices: *χ*^2^(39) = 83.03, *p* < .001, *TLI* = .96, *CFI* = .98, *RMSEA* = .06, *SRMR* = .03.

#### 2.2.5 Barriers to and reasons against DM use in PE

In addition to the quantitative questions, the students were asked an open question at the end of the questionnaire about barriers to and reasons against the use of DM in PE.

### 2.3 Design and procedure

#### 2.3.1 Pre-study organization

Ethical approval was obtained from the local universities' ethics committee. The questionnaire was then piloted by two 16-year-old students and subsequently discussed with the students in order to check the questionnaires general comprehensibility and basic suitability, specifically to identify potential linguistic errors and problems with the questionnaire's congruence. No changes were made because the questionnaire appeared to be suitable in terms of its feasibility and suitability for the target group. Contact lists from prior research with PE teachers and personal contacts to PE teachers were used to acquire potential participants. PE teachers were contacted via e-mail with information about the study's purpose and procedure. Teachers interested in the study were asked to respond to the e-mail to make further arrangements (e.g., scheduling of data collection, information letter for parents), which was done via e-mail or telephone calls. After the PE teachers and school principals agreed to participate in the study, the questionnaire was provided to the teachers as an online questionnaire via Unipark that could be forwarded to the students in the form of a link or QR code.

#### 2.3.2 Measurement organization

The teachers provided sufficient time (approximately 15 min) in their PE lessons for the students to complete the questionnaire on their mobile devices. At the beginning of the questionnaire, the students were informed of the purpose of the survey, the anonymization of the data collection and evaluation, the voluntariness of participation, the possibility of making queries (including contact information), and that they should answer the questionnaire alone based on their personal opinions. The questions were ordered in the same manner as in Section 2.2. After the survey was completed, the answers were transmitted digitally via Unipark to the study organization, and the teachers conducted their PE lessons as usual.

### 2.4 Data analysis

The data were processed using IBM SPSS Statistics version 29. The data were prepared by screening for outliers, missing values, and implausible answers (i.e., removing participants who marked the same value for each questionnaire item). To answer the RQs, descriptive statistics and inferential statistics with an alpha level at *p* < .05 were calculated. The prerequisites for the inferential statistics were checked according to Tabachnick and Fidell (41).

RQ1 was answered with descriptive statistics (*M*, *SD*, *Min*, *Max*, and frequency) on students' usage intentions regarding the five different types of DM use.

RQ2 was answered with an analysis of variance between the five different types of DM use—with age, gender, and the amount of DM use during leisure time as covariates. The assumptions for the analysis of variance were checked. Boxplots for all measurement points showed three participants with extreme values for the entertainment item. As the outliers were neither unusual values nor measurement errors that appeared implausible, they were left in the analysis. The data for the five different types of DM use were not normally distributed—particularly the entertainment item, which showed considerable left-skewness. However, due to the size of the sample and the central limit theorem, this requirement violation did not preclude the execution of the variance analysis (42). The assumption of homoscedasticity was violated (Mauchly test for sphericity: *p* < .001; Greenhouse-Geisser Epsilon = .924). For this reason, the Huynh-Feldt correction was used. Subsequently, Bonferroni *post hoc* tests were carried out for pairwise comparisons to determine where the differences between the types of DM use occurred.

RQ3 was answered with a multiple linear regression (enter method) to analyze whether and to what extent the types of behavioral regulation as well as age, gender, amount of DM use during leisure time, and students' experiences with DM use in PE predict students' intention for future DM use in PE. The assumptions for the multiple linear regression were checked. The Durbin-Watson statistic (*d* = 1.97) verified the independence of residuals. Linearity and homoscedasticity were assessed via visual inspection of plots of studentized residuals against unstandardized predicted values. No multicollinearity was detected. While some predictors, such as external regulation, showed moderate to strong right-skewness, the regression residuals were normally distributed (confirmed via visual inspection). Therefore, skewness in the predictors did not affect the regression analysis. Potential outliers were identified with studentized residuals, leading to one potential outlier whose residuals were above ±3 SDs. However, as in RQ2, a potential outlier was included in the regression analysis, as the data did not result from measurement errors and did not appear implausible. Risky leverage values and Cook's distance values indicated no further potential outliers.

The qualitative data on reasons against DM use in PE served as additional information to interpret quantitative findings. The categories were inductively developed from the data by the first author using a qualitative content analysis (43). To enhance credibility, the identified categories were discussed with the second author based on the original quotations. Frequencies were calculated and selected quotations were used to explain the identified categories.

## 3 Results

### 3.1 Background information: Students' DM use during leisure time, at school, and in PE

To provide background information for interpreting the results of the RQs, students were asked about their DM use during leisure time, at school, and in PE. The sample had a self-reported average digital screen time of 4.49 h (*SD* = 2.04) during leisure time. Smartphones (97%, *n* = 281), tablets (44%, *n* = 127), and smart TVs (25%, *n* = 73) were the most commonly used devices. WhatsApp (77%, *n* = 244), Snapchat (68%, *n* = 199), and Instagram (63%, *n* = 182) were the most commonly used applications during leisure time. Students used DM daily for communication (86%, *n* = 251), entertainment/streaming (69%, *n* = 200), and relaxation/recovery (40%, *n* = 115). The frequency of DM use differed among school subjects. DM were most frequently used in informatics/computer science and least frequently used in PE (see Table 1). The descriptive statistics for DM use in PE (see Table 2) show that DM was most frequently used for entertainment purposes (e.g., music during exercises; 39% often/always) and not at all or rarely to occasionally for other purposes.

**Table 1:** Descriptive statistics on DM use in different school subjects.

Subjects	*M*	*SD*	*Min*	*Max*
Arts (*n* = 237)	2.80	1.68	1	6
Foreign languages (*n* = 288)	3.73	1.84	1	6
German (*n* = 291)	3.45	1.94	1	6
Informatics/Computer science (*n* = 136)	4.94	1.72	1	6
Mathematics (*n* = 291)	3.48	1.90	1	6
Music (*n* = 177)	3.62	1.91	1	6
Natural sciences (*n* = 289)	3.51	1.87	1	6
Physical education (*n* = 286)	1.74	1.12	1	6
Religion/Ethics/Philosophy (*n* = 237)	3.43	1.89	1	6
Social science subjects (*n* = 288)	4.07	1.54	1	6

Based on a Likert scale from “never” (1) to “always” (6).

**Table 2:** Descriptive statistics on current DM use in PE of different types of DM use.

Current DM use in PE (type of DM use)	*M* (*SD*)	*Min-Max*	1 never	2	3	4	5	6 always
Instruction (e.g., videos on correct exercise execution)	2.20 (1.36)	1–6	42.3%	22.7%	18.2%	9.3%	4.5%	3.1%
			*n* = 123	*n* = 66	*n* = 53	*n* = 27	*n* = 13	*n* = 9
Feedback/correction (e.g., filming exercise execution)	2.14 (1.50)	1–6	51.5%	16.5%	13.4%	7.9%	5.8%	4.8%
			*n* = 150	*n* = 48	*n* = 39	*n* = 23	*n* = 17	*n* = 14
Organization of lessons (e.g., drawing lots for teams)	1.78 (1.40)	1–6	67.7%	13.4%	3.8%	7.2%	4.1%	3.8%
			*n* = 197	*n* = 39	*n* = 11	*n* = 21	*n* = 12	*n* = 11
Knowledge transfer (e.g., background knowledge about sports)	1.73 (1.11)	1–6	60.1%	19.9%	10.7%	6.2%	2.1%	1.0%
			*n* = 175	*n* = 58	*n* = 31	*n* = 18	*n* = 6	*n* = 3
Entertainment (e.g., music during exercises)	3.60 (1.79)	1–6	19.2%	13.1%	14.1%	14.4%	20.3%	18.9%
			*n* = 56	*n* = 38	*n* = 41	*n* = 42	*n* = 59	*n* = 55

*n* = 291.

### 3.2 Research question 1: Students' acceptance (i.e., intention for future use) of different types of DM use in PE

**Figure 2:**
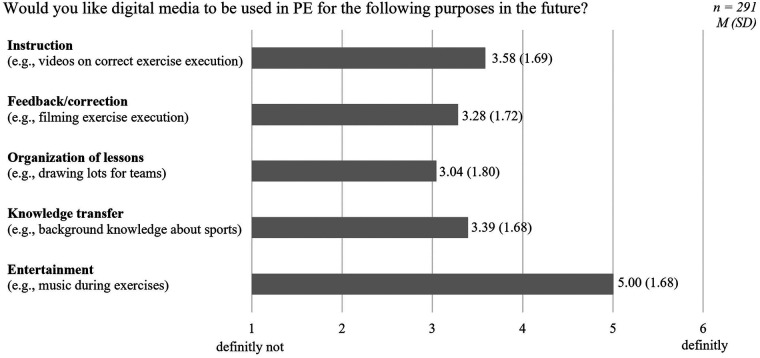
Students' acceptance of different types of digital media use in physical education.

Table 3 shows the descriptive data (*M*, *SD*, *Min*, *Max*) of the students' intentions regarding the five different types of DM use in PE. Figure 2 visualizes the strength of students' intention regarding the different types of DM use in PE. The highest usage intention was for entertainment, with three-quarters of the participants stating that they would use DM for entertainment purposes in future PE lessons. The lowest usage intention was for the organization of lessons, with almost half of the participants not wanting to use DM for organizational purposes in future PE lessons. Regarding DM use for the other three purposes (instruction, feedback/correction, and knowledge transfer), most students were undecided.

**Table 3:** Descriptive statistics on the intention for future DM use of different types of DM use.

Intention for future DM use in PE (type of DM use)	*M* (*SD*)	*Min-Max*	1 definitely not	2	3	4	5	6 definitely
Instruction (e.g., videos on correct exercise execution)	3.58 (1.69)	1–6	16.8%	10.7%	19.9%	22.3%	11.0%	19.2%
			*n* = 49	*n* = 31	*n* = 58	*n* = 65	*n* = 32	*n* = 56
Feedback/correction (e.g., filming exercise execution)	3.28 (1.72)	1–6	21.6%	13.1%	24.1%	13.1%	13.1%	15.1%
			*n* = 63	*n* = 38	*n* = 70	*n* = 38	*n* = 38	*n* = 44
Organization of lessons (e.g., drawing lots for teams)	3.04 (1.80)	1–6	29.9%	14.8%	15.8%	14.8%	10.3%	14.4%
			*n* = 87	*n* = 43	*n* = 46	*n* = 43	*n* = 30	*n* = 42
Knowledge transfer (e.g., background knowledge about sports)	3.39 (1.68)	1–6	18.9%	13.1%	21.6%	18.6%	12.0%	15.8%
			*n* = 55	*n* = 38	*n* = 63	*n* = 54	*n* = 35	*n* = 46
Entertainment (e.g., music during exercises)	5.00 (1.68)	1–6	11.0%	2.4%	3.8%	5.2%	13.4%	64.3%
			*n* = 32	*n* = 7	*n* = 11	*n* = 15	*n* = 39	*n* = 187

*n* = 291.

### 3.3 Research question 2: Differences in students' intentions for future use between types of DM use in PE

To answer the second RQ, an analysis of variance of students' intentions regarding different types of DM use in PE, with age, gender, and the amount of DM use during leisure time as covariates was conducted (see Table 4). The analysis of variance revealed a significant main effect regarding the types of DM use [*F*(3.79, 1,087.90) = 6.71, *p* < .001, partial *η*^2^ = .023], indicating a small effect. Moreover, significant interaction effects with the intention for the different types of DM use were found regarding the covariates of age [*F*(3.79, 1,087.90) = 4.98, *p* < .001, partial *η*^2^ = .017] and gender [*F*(3.79, 1,087.90) = 7.37, *p* < .001, partial *η*^2^ = .025], both showing small effects as well. This indicates that the intention to use different types of DM in PE varied with age and gender. The interaction between DM use type and DM use amount during leisure time was not significant [*F*(3.79, 1,087.90) = 0.27, *p* = .887, partial *η*^2^ = .001].

**Table 4:** Analysis of variance on the intention of different types of DM use with covariates.

Sources	Type III sum of squares	df	Mean square	F	p-value	Partial eta-square
Types of DM use	48.57	3.79	12.81	6.71	<.001*	.023
Types of DM use * age (covariate)	36.04	3.79	9.51	4.98	<.001*	.017
Types of DM use * gender (covariate)	53.36	3.79	14.08	7.37	<.001*	.025
Types of DM use * amount of DM use in leisure time (covariate)	1.97	3.79	0.52	0.27	.887	.001
Error	2077.71	1087.90	1.91			

*n* = 291; Huyn-Feldt correction reported.

* significant on 5% alpha error level.

Bonferroni-adjusted *post hoc* tests (see Table 5) show that the students' intention to use DM for entertainment (*M* = 5.00, *SD* = 1.68) was significantly higher than in all other types of DM use: instruction (*M* = 3.58, *SD* = 1.69), feedback (*M* = 3.28, *SD* = 1.72), organization (*M* = 3.04, *SD* = 1.79), and knowledge transfer (*M* = 3.39, *SD* = 1.68). Moreover, students' intention to use DM for instruction (*M* = 3.58, *SD* = 1.69) was significantly higher than for feedback (*M* = 3.28, *SD* = 1.72) and organization (*M* = 3.04, *SD* = 1.79). Another significant difference was that students' intention to use DM for knowledge transfer (*M* = 3.39, *SD* = 1.68) was significantly higher than for organization (*M* = 3.04, *SD* = 1.79).

**Table 5:** Bonferroni-adjusted pairwise comparisons of intentions for different types of DM use.

Comparison	Mean difference	SE	p	95% CI
Instruction vs. feedback	.30	.10	.032*	(.02, .58)
Instruction vs. organization	.54	.12	<.001*	(.19, .88)
Instruction vs. knowledge transfer	.19	.09	.360	(−.06, .44)
Instruction vs. entertainment	−1.43	.11	<.001*	(−1.74, −1.11)
Feedback vs. organization	.24	.12	.391	(−.09, .57)
Feedback vs. knowledge transfer	−.11	.11	1.00	(−.41, .19)
Feedback vs. entertainment	−1.72	.12	<.001*	(−2.05, −1.39)
Organization vs. knowledge transfer	−.35	.11	.017*	(−.66, −.04)
Organization vs. entertainment	−1.96	.13	<.001*	(−2.32, −1.60)
Knowledge transfer vs. entertainment	−1.61	.12	<.001*	(−1.94, −1.28)

*n* = 291.

* significant on 5% alpha error level.

Due to the significant interaction effects with the covariates of gender and age, subsequent t-tests to compare younger students (14–15 years old) and older students (16–18 years old) and between male and female students were conducted for every type of DM use. In terms of age, usage intention was weaker among younger students than among older students for four out of five types of DM use: instruction [14–15: *M* = 3.31, *SD* = 1.68; 16–18: *M* = 3.78, *SD* = 1.68; *t*(289) = −2.34, *p* = .010], feedback/correction [14–15: *M* = 2.82, *SD* = 1.61; 16–18: *M* = 3.68, *SD* = 1.71; *t*(289) = −4.13, *p* < .001], knowledge transfer [14–15: *M* = 3.04, *SD* = 1.71; 16–18: *M* = 3.66, *SD* = 1.62; *t*(289) = −3.14, *p* < .001], and entertainment [14–15: *M* = 4.74, *SD* = 1.82; 16–18: *M* = 5.20, *SD* = 1.55; Welch: *t*(241.53) = −2.25, *p* = .013]. Regarding gender, t-tests for every type of DM use in PE revealed a significant difference between male (*M* = 4.54, *SD* = 1.86) and female (*M* = 5.42, *SD* = 1.39) students in the intention to use DM for entertainment [Welch: *t*(252.28) = 4.51, *p* < .001]. Male and female students did not differ significantly in their intention to use other types of DM in PE.

### 3.4 Research question 3: Predictors of students' intention to use DM in PE

To examine whether types of behavioral regulation (see Table 6 for descriptive results) predict students' intention to use DM in PE, a multiple linear regression analysis using the enter method was conducted. The regression results (see Table 7) show that behavioral regulation statistically significantly predicts students' intention to use DM in PE [*F*(10, 259) = 29.242, *p* < .001]. The model explains 51% of the variance in the independent variable (adj. *R*^2^ = .51). Identified regulation (ß = .337, *p* < .001), intrinsic motivation (ß = .264, *p* < .001), introjected regulation (ß = −.106, *p* = .049), and amotivation (ß = −.155, *p* = .003) were found to be significant predictors for students' intention to use DM in PE. The other types of behavioral regulation, age, gender, amount of DM use during leisure time, and experiences with DM use in PE were not significant predictors of students' intention to use DM in PE.

**Table 6:** Descriptive statistics on behavioral regulation regarding future DM use.

Behavioral regulation	*M*	*SD*	*Min*	*Max*
Self-determined regulation	2.99	1.28	1	6
Intrinsic motivation	2.91	1.41	1	6
Integrated regulation	2.61	1.41	1	6
Identified regulation	3.45	1.54	1	6
Controlled regulation	2.15	1.03	1	6
Introjected regulation	2.06	1.15	1	6
External regulation	1.89	1.39	1	6
Amotivation	2.50	1.47	1	6

*n* = 274; based on a Likert scale from “does not apply at all” (1) to “fully applies” (6).

**Table 7:** Multiple linear regression analysis for intention for future DM use.

Predictors	ß	SE	T	p	adj. R^2^
Intrinsic motivation	.264	.06	3.42	<.001*	
Integrated regulation	.142	.06	1.90	.059	
Identified regulation	.337	.04	5.80	<.001*	
Introjected regulation	−.106	.05	−1.97	.049*	
External regulation	.085	.04	1.52	.131	
Amotivation	−.155	.04	−2.99	.003*	
Age	.024	.05	.51	.612	
Gender	.003	.10	.08	.940	
Amount of DM use in leisure time	−.035	.02	−.79	.431	
Experience in DM use in PE	.086	.05	1.83	.069	
Total					.51

*n* = 274.

* significant on 5% alpha error level.

### 3.5 Additional information: Barriers to and reasons against the use of DM in PE

To provide background information for interpreting the results of the RQs, the qualitative statements of the students on the barriers to and reasons against using DM in PE were evaluated. Out of 291 students, 149 (51%) named barriers to or reasons against the use of DM in PE. Thirty-one students mentioned more than one aspect, resulting in 185 individual statements. The qualitative analysis revealed eight categories of barriers to or reasons against the use of DM in PE (see Table 8).

**Table 8:** Barriers and reasons against the use of DM in PE.

Category	Frequency	Example quotes
Distraction and loss of focus in PE	*n* = 57; 30.8%	“You could get distracted and concentrate less on the sport.”
		“You can do other things with smartphones, for example, that can distract you.”
Loss of physical activity in physical education	*n* = 41; 22.2%	“Media would reduce the amount of time we spend doing sport and we already have too little active time.”
		“People would spend more time looking on content on screens than doing sports.”
Discrepancy between digitalization and sports/physical education	*n* = 21; 11.4%	“Digital media are actually the opposite of sport.”
		“It's about physical exercise, not playing on devices.”
No/limited benefits of digital media in physical education	*n* = 20; 10.8%	“In my opinion, you dońt need digital media in physical education lessons.”
		“Face to face learning is better.”
Digital overload in daily life	*n* = 20; 10.8%	“You are on your phone all day anyway, so I dońt need any more in class.”
		“Many people are on their smartphones all day anyway.”
Insecurities regarding data protection	*n* = 13; 7%	“Videos or images could be sent to others inappropriately.”
		“Others could be accidentally filmed in the background.”
Loss of social interaction with peers and teachers in physical education	*n* = 8; 4.3%	“Teachers can teach me better than YouTube.”
		“Maybe people won't talk to each other anymore.”
Problems with technical equipment and much time effort	*n* = 5; 2.7%	“Because there are usually problems with the internet in the hall.”
		“Because then all students will be dependent on digital devices.”

*n* refers to the number of statements, % refers to the proportion in relation to the total number of statements; examples are translated to English from German.

## 4 Discussion

The present study aimed to investigate students' acceptance (defined as their intention to use) of different types of DM use in PE. The results show that only one type of DM use (entertainment) is strongly accepted, while the other four types of DM use in PE (instruction, feedback, organization, and knowledge transfer) are only moderately accepted by students. The acceptance of DM use in PE interacts with age and gender in some types of DM use. Identified regulation and intrinsic motivation were found to be positive predictors of students' acceptance of DM use in PE in general, whereas introjected regulation and amotivation were found to be negative predictors.

Overall, the students' acceptance of DM use in PE was moderate, particularly in terms of instruction, feedback, organization, and knowledge transfer. Equal numbers of students were in favor of and against the use of DM in PE, which indicates ambivalence toward the use of DM in PE among the students. For those in favor, it can be assumed that they like to transfer DM into PE because of the potential and advantages DM can bring to the traditional learning environment of PE. This can be explained by our findings on the predictors of acceptance, showing that identified regulation (i.e., usefulness) is the strongest predictor of DM acceptance in PE. Other studies have also shown that it is particularly important for the acceptance of DM that students see a benefit in the use of DM [e.g., (38)]. Moreover, aspects from research on motivation in PE can explain why students may like the integration of DM in PE. White et al. (39) found that especially novelty, choices and challenges are important for self-determined motivation in PE. DM are seen to bring those potential into PE [e.g., gamified elements: (4, 5)].

In contrast, the students who were against the use of DM in PE may not reject DM *per se*, but rather perceive the use of DM for physical activity/sports as inappropriate or useless. This assumption is supported by the way the students in our study use DM during leisure time. The students mainly used DM during leisure time for communication, entertainment, and relaxation purposes, which are mainly sedentary and physically inactive forms of DM use (44). These forms of DM use may be in contrast with the traditionally physically active subject of PE (45). In addition, the low acceptance of some students on the use of DM in PE can be supported by the findings of Jones et al. (46), which showed that although students have grown up with DM and use them in many contexts, they do not automatically have positive attitudes toward DM in all contexts.

Another explanation for the ambivalence toward the use of DM in PE in the group of students may be the students' prior different experiences with DM use in PE. In our sample, PE was the subject in which DM was used the least—i.e., compared to other subjects, such as informatics and mathematics. Specifically, prior experiences with different types of DM use in PE are low to moderate—with means between 1.73 and 3.60 on a scale ranging from 1 (never) to 6 (always). Therefore, many students may be unfamiliar with the use of DM in PE, which may make it difficult for them to imagine using DM in PE. In addition to the quantity of DM use, the quality of DM use experiences may also be important for students' acceptance (47). Regarding students' prior experiences it can be assumed that the quality of the DM use in PE differ in terms of usability, relevance, or potential for engagement. Therefore, students' ratings of the quality of their experiences may vary widely—from positive to negative, helpful to disturbing, and fun to boring—and thus lead to ambivalence in the group of students toward the acceptance (i.e., intention to use DM in PE).

Our qualitative results can provide further explanations for the students' rather moderate acceptance of DM use in PE. These qualitative results revealed eight categories of barriers to and reasons against DM use in PE, which can be structured into three overarching areas: discrepancy between DM and sports, the lack of any real benefit, and technical issues. First, regarding discrepancy, the students stated that DM are incompatible with the physical nature of sports/PE (e.g., “Digital media are actually the opposite of sports”) and can lead to less time to move (e.g., “People would spend more time looking at content on screens than doing sport”). This perspective has also been presented in prior research on students' acceptance of DM in PE, where the loss of time to move was mentioned as a negative aspect of DM use in PE (35). The loss of exercise time can be attributed to concerns about distraction by DM (e.g., “You could get distracted and concentrate less on the sport”) and time lost due to technical obstacles (e.g., “Because there are usually problems with the internet in the hall”). Second, regarding the lack of any real benefit of using DM in PE, the students mentioned both social components and the learning processes in PE (e.g., “Teachers can teach me better than YouTube”). In terms of social aspects, Killian and Woods (36) found that students experienced reduced interaction with teachers in PE when DM were used. Besides the interaction with teachers, students are also concerned that DM would lead to less interactions with classmates, as highlighted in the quote “Maybe people won't talk to each other anymore”. On a theoretical level both, less interaction with teachers and classmates, can be linked to the relevance of the basic psychological need for relatedness in the SDT (12). Satisfaction of the need for relatedness (e.g., in cooperative tasks) is important for self-determined motivation, while need frustration is associated with external regulation and amotivation (19, 39). Regarding learning processes, our findings contradict those of previous studies in which students positively evaluated DM use for movement and learning tactics (34, 35). These findings emphasize that the purpose and the intended usefulness have to be clearly communicated to the students, as a lack of usefulness results in meaningless use of DM, which may lead to amotivation. Third, regarding technical issues, students mentioned equipment (e.g., “Because there are usually problems with the internet in the hall”) and data protection (“Videos and images could be sent to others inappropriately”). Both issues have been raised in other studies: Problems with the Wi-Fi were discussed in the study of Koh et al. (35), and prohibited filming by classmates was mentioned by Greve et al. (34). In particular, the fear of being filmed/photographed by classmates can be associated with shame about the own body, which can be a major problem with negative effects on motivation in PE (39). In sum, the qualitative results reveal diverse concerns of the students regarding DM use in PE, underscoring a key finding in the previous paragraph: students' ambivalence toward the use of DM in PE. The ambivalence is on the one hand shown in the amount 51% of the 291 students (i.e., the half of the students), who have answered the facultative question on barriers to or reasons against DM use in PE. On the other hand, the heterogeneity of the mentioned barriers (including learning, social, technical aspects) also indicated that students evaluate the use of DM in PE very differently.

Regarding the different types of DM use, students accepted DM for entertainment the most and for organization the least. Although the differences between the types of DM use are statistically significant, their practical significance is limited, as indicated by the low effect size ($\eta_{p}^{2}=.023$). Only the difference between entertainment and the other types of DM use appear to be relevant, as they are clearly reflected in the confidence intervals and at the descriptive level. The strong acceptance of DM for entertainment can be explained by two central arguments. First, our sample already used DM extensively for entertainment purposes, with 70% using it daily. This high usage aligns with previous findings [e.g., (48, 49)] and highlights the strong presence of DM use for entertainment in adolescents' lives. The high level of acceptance of DM for entertainment in PE may therefore stems from familiar DM use routines, rather than a perceived usefulness for PE itself. Second, the students' experienced the use of DM in PE for entertainment more often (mean of 3.60) compared to the other types of DM use (means of 1.73–2.20). This greater experience with the entertainment purpose likely helped the students to imagine this type of DM use better than the others.

In addition to the generally high acceptance of DM for entertainment, we found that female students accepted this type of DM use more than male students. This result contradicts other findings of technology acceptance research in the educational context, which show that boys tend to accept DM in general more than girls (29, 33). However, this discrepancy to other findings can be explained by the operationalization of “entertainment”, which, due to sociocultural differences, probably appealed more to girls than boys. While previous studies like Bourgonjon et al. (29) and Sun and Law (33) measured students' acceptance of digital video games in education, which are more pronounced among male students (50), our study cited listening to music as an example of entertainment. Existing literature shows that music consumption in general is more pronounced among female students (51) as well as activities like dancing combining music with sports (52). The difference regarding gender is small according to the effect size ($\eta_{p}^{2}=.025$) but nevertheless shows that personal preferences and sociocultural explanations might play a role. Given this small effect size, the gender-specific differences should not be seen as confirmation of gender-specific stigmas, but rather as an indication that “listening to music” is only one example of entertainment. Other entertainment purposes of DM in PE [e.g., gamification: (4, 5)], which are more related among male students like playing-video-games (50) might change the gender difference in acceptance. Gender differences should be examined more closely in the future to discuss how gender media practice may interact with the students' acceptance of DM in PE.

In comparison to the strong acceptance of entertainment, the rather low acceptance of DM use for instruction, feedback, knowledge transfer, and (in particular) organization can be explained in two ways. First, these types of DM use were less familiar to the students than entertainment, both in PE and during leisure time, and more difficult for the students to imagine than entertainment. Second, it can be assumed that the benefits of the types of DM use (instruction, feedback, knowledge transfer and organization) were probably not clear enough to the students. This may have been particularly the case for organizational DM use, as the students may not have perceived direct added value from the example in the questionnaires “drawing of teams”, which would primarily benefit teachers.

Regarding age older students were more likely to accept the use of DM in PE, whereby a small effect size ($\eta_{p}^{2}=.017$) limits the practical relevance here as well. Nevertheless, these age differences may result of developmental factors or learning processes, such as increased media experiences in leisure time and school. Regarding the DM use in leisure time, adolescents spend more time using DM the older they get (8). At school, secondary education teachers are more likely to use DM than primary education teachers (53, 54), which influences the frequency of DM use so that DM is more used in higher grades. Both, the DM use in leisure time and in school, may lead to more experiences and a more complex understanding of the potential of DM for PE. The complexity of the potential of DM in PE is reflected by Jastrow et al. (2) showing, that DM can not only be used for traditional topics like movement instruction but also for media-critical topics about content on social media, which may be more relevant for older students.

Identified regulation (i.e., usefulness) and intrinsic motivation (i.e., enjoyment) positively predict students' acceptance (i.e., behavioral intention) of DM in PE. Identified regulation had the strongest positive association with students' acceptance in PE (ß = .337), followed by intrinsic motivation (ß = .264). These results highlight the crucial role of identified regulation (i.e., usefulness) and intrinsic motivation (i.e., enjoyment) in explaining intention align with prior technology acceptance research in educational settings in general [e.g., (26)] and in PE classes in particular. Koh et al. (35) and Merino-Campos et al. (43) lead as the main argument that students are more likely to accept DM in PE if they perceive added value, such as support in the learning process. This fits to the strongest prediction of behavioral intention by identified regulation and aligns with the theoretical assumptions of Deci and Ryan (12), which highlight how identified regulation as an self-determined type of motivation is characterized by people behaving out of the underlying value of a behavior. Our finding on intrinsic motivation is consistent with those of Merino-Campos et al. (37), who found that the more students enjoy using DM, the lower their resistance to the use of DM in PE. Additionally, the results regarding enjoyment align with Roth et al. (38), who highlighted the connection between positive affect and fostering students' intention to use DM.

Amotivation (i.e., meaninglessness) and introjected regulation (i.e., social expectations/norms) are negatively associated with acceptance, but both associations are weaker than those of identified regulation and intrinsic motivation, highlighting the relevant role of self-determined motivation in PE. The statistical effect of amotivation (ß = −.155) is stronger than for introjected regulation (ß = −.106), indicating that perceiving DM as meaningless is more negative for students' than using DM in PE because of social pressure/norms. In contrast to identified regulation (i.e., usefulness) and intrinsic motivation (i.e., enjoyment), amotivation (i.e., meaninglessness) and introjected regulation (i.e., social expectations/norms) have received less attention in recent research on students' acceptance of DM in PE. Nevertheless, the results can be discussed against the background of SDT. Amotivation can be interpreted as the opposite of usefulness, as a behavior is amotivated when it lacks efficacy (12) and thus represents a relevant counterpoint to the strongly positive explanation of acceptance by identified regulation (ß = .337). Moreover, amotivation is also reflected in the qualitative results of our study in the category “no real benefits” in terms of barriers to and reasons against DM in PE. In terms of social expectations/norms, our findings reveal that a strong introjection of social rules and expectations (12) correlates negatively with students' intention to use DM in PE. This finding on the one hand contradicts the finding of Granić (22), which showed that subjective norms are positive predictors for usefulness of education technology and on the other hand it aligns with findings in PE showing that a high media norm (defined as social influence) decreases perceived usefulness and increases concerns about the match between DM and PE (38).

### 4.1 Strengths and limitations

Our study has four major strengths highlighting our study as an important addition to the current state of research on students' acceptance of DM in PE. First, we focused on the perspectives of students, who constitute an important but currently underrepresented stakeholder group in research on technology acceptance in PE. Second, we surveyed the students prospectively regarding their acceptance of DM use in PE and were thus able to map their usage intentions for future use. Third, we looked at students' acceptance of different types of DM use in PE. Fourth, our research is theoretically conceptualized considering aspects of technology acceptance research and provides an opportunity to link the technology acceptance research to SDT as a popular and suitable theory regarding motivation in PE (19, 39).

Our study has limitations related to its sample, design and measures/measurement organization. First, our sample is relatively homogeneous, which leads to a limited generalizability. The homogeneity of the population is i.e., shown in age (14–18-years), educational track (grammar level) and cultural background (urban areas in Germany). Results may differ in other educational settings (e.g., primary education, vocational schools), where the target group might have other attitudes towards the use of DM in PE. Moreover, results are only limited transferable to other cultural or national contexts, where the structure of the school system might differ substantially (e.g., because of other digital equipment). Additionally, the sample is limited to binary gender (male/female), which is a limitation in that those students with other or no gender attributions were not considered. Second, we used a cross-sectional design, which allowed us to statistically explain the students' acceptance of DM via predictors (i.e., behavioral regulation), but it did not allow us to make any statements about causality regarding the influence of behavioral regulation on acceptance. Third, the used measures respectively the measurement organization can be seen as limitations. Regarding the measure we used self-reports, which carry a risk for recall bias (e.g., related to experiences with and amount of DM use) and social desirability (e.g., related to intention to use DM). However, self-reported measures are important for obtaining information about students' experiences and perceptions. Moreover, social desirability could be enhanced in that the teachers were present during the answering of the questionnaire by the students. In addition, we measured acceptance as the students' intention for different types of DM use in PE with single items. Although the single item measures were appropriate due to their specificity and high measurement economy in terms of time and practicability, they have psychometric limitations regarding reliability and validity (55). Moreover, the definition of acceptance in a narrow sense as students' intention to use have to be discussed in the context of PE practice. The actual use of DM in PE does not depend solely on the students' usage intention. Instead external factors like institutional constraints (e.g., availability of technical equipment), teacher behavior (e.g., teachers' self-efficacy) and curricular demands (e.g., regulations on topics and purposes of DM use), should also be considered. Regarding measurement organization, the limited time available to the students to complete the questionnaire (approximately 15 min) may have led them to rush through their answers, particularly in the case of the open questions at the end of the questionnaire.

### 4.2 Empirical, practical and systemic implications

Empirical, practical and systemic implications can be derived from our study. Regarding empirical implications our study highlights four central points for future research. First, further research with different subgroups is needed to make differentiated statements for different student groups. These subgroups could refer to school type, age groups/grades, regions/country or experiences of students in DM use. Second, longitudinal study designs are needed to better investigate the causal relationships between behavioral regulation, intention to use, and future DM use in PE. Comparing students' intention to use DM and their evaluations of DM use afterwards could be interesting (i.e., a comparison between the expected benefit of use and the actual benefit of use). Third, besides the intention of DM use in PE as a prospective component of acceptance, the acceptance of actual DM use in PE should be investigated. Therefore, objective measures (e.g., observations or log-based data) can be seen as an addition to a questionnaire to gain more information about the use of DM in PE and e.g., the effect of DM use in PE on students' movement-behavior. Fourth, the results regarding the acceptance (i.e., students' intention for the future DM use in PE) can be used to design interventions to enhance students' motivation in PE. Prior research on interventions to enhance students' motivation in PE shows that the acceptance of DM by students is relevant for reaching effective interventions (e.g., 56). Therefore, our results regarding accepted types of DM use and predictors of acceptance should be utilized in the design of future DM interventions. In addition to this, considerations on classification systems regarding motivational behavior by teachers (e.g., 57) could be transferred to the use of DM in PE during the design and evaluation of the interventions.

Our findings have two practical implications for PE. First, teachers should consider students' acceptance of different types of DM use (i.e., instruction, feedback, organization, knowledge transfer, and entertainment) in their planning of DM use in PE. This means that teachers who want their students' to like PE with DM should think carefully about why they are using DM and not just assume that students will automatically prefer digital PE lessons to analogue ones. Therefore, teachers should focus on the usefulness of DM (i.e., students should see a benefit in using DM in PE) and on the enjoyment of DM use (i.e., students should enjoy using DM in PE) as relevant predictors of students' general DM acceptance in PE. This consideration of students' acceptance of DM in PE should not lead to a neglect of other pedagogical aspects (e.g., movement quality). Instead, taking into account students' acceptance of DM in PE should lead to the potential offered by DM for achieving educational goals in physical education (2) being exploited. Moreover, as the second practical implication, teachers should take students' multiple concerns about DM use in PE seriously, including the discrepancy between the use of DM and doing sports/being physically active, the lack of any real benefit of DM in PE, and technical issues related to DM use in PE. In addition, teachers should be prepared to deal with technical hurdles (e.g., preparing Plan-B solutions and sensitizing students for data security aspects). Plan-B solutions could include using apps that can be used offline in case of possible internet outages or carry analog worksheets with them as an alternative. In order to sensitize students' awareness of data security aspects, one idea would be to address their concerns (e.g., sending pictures to third parties) before the use of DM in PE and to point out possible consequences of neglecting data security.

On the systemic level our study leads to two implications. First, the findings are not only directly relevant to PE teachers but are also relevant to multipliers in teacher education at universities and in-service training. These multipliers and PE teachers might benefit significantly from the use of DM for organizational purposes, while students in PE do not have a really strong intention to use them in this regard. Therefore, teacher education at universities and in-service training should integrate formats of critical reflection on the use of DM in PE so that teachers are able to combine the opportunities and challenges DM bring to PE successfully. Second, the results should be considered on a systemic level in terms of curriculum design. Curricula serve as guidelines for teachers in their structural planning of their teaching and are increasingly integrating digital aspects as cross-cutting themes. However, it should be considered that the use of DM in the specific context of PE might not be a guarantee of enhanced learning experiences. Therefore, PE teachers should continue to have the freedom to use DM in PE, but should not be obliged to.

## 5 Conclusion

We investigated in our study students' acceptance (defined as intention to use DM in PE) of different types of DM use in PE (i.e., instruction, feedback, organization, knowledge transfer, and entertainment) and analyzed the acceptance in relation to students' motivation to use DM in PE according to OIT as a theoretical framework. We found that students' acceptance of DM in PE was generally moderate, with a high standard deviation characterizing ambivalence in the sample (i.e., as many students with high acceptance as students with low acceptance). Regarding the different types of DM use in PE, only entertainment was strongly accepted; the other types were moderately accepted by students. To explain students' ambivalence toward and rather moderate overall acceptance of DM in PE, students' concerns regarding the use of DM in PE must be considered. The concerns arise in terms of a discrepancy (i.e., different meanings) between DM and physical activity/sports, the lack of any real benefit of DM in PE, and technical issues related to DM use in PE. Regarding the predictors of students' acceptance of DM in PE, usefulness (i.e., identified regulation) and enjoyment (i.e., intrinsic motivation) predict acceptance positively, while social expectations (i.e., introjected regulation) and meaninglessness (i.e., amotivation) predict acceptance negatively. Future research should further explore students' acceptance of DM in PE, especially research with other samples (e.g., other countries and cultural background, other school types) and longitudinal research investigating causal effects regarding usage intention and actual use. As a practical recommendation, PE teachers should consider students' different intentions regarding different types of DM use and focus on the usefulness and enjoyment of DM use in PE as well as facing students' concerns seriously. This consideration of students' acceptance can enable both, a successful and a critical integration of the diverse potential that DM can bring to PE. Thereby, it seems necessary to establish a dialogue between teachers' educational goals, schools' technical equipment and students' acceptance of DM in PE.

## Data Availability

The raw data supporting the conclusions of this article will be made available by the authors, without undue reservation.
